# Genetic Variants of the Brain-Derived Neurotrophic Factor and Metabolic Indices in Veterans With Posttraumatic Stress Disorder

**DOI:** 10.3389/fpsyt.2018.00637

**Published:** 2018-11-27

**Authors:** Lucija Tudor, Marcela Konjevod, Matea Nikolac Perkovic, Dubravka Svob Strac, Gordana Nedic Erjavec, Suzana Uzun, Oliver Kozumplik, Marina Sagud, Zrnka Kovacic Petrovic, Nela Pivac

**Affiliations:** ^1^Laboratory for Molecular Neuropsychiatry, Division of Molecular Medicine, Rudjer Boskovic Institute, Zagreb, Croatia; ^2^Department of Biological Psychiatry and Psychogeriatry, University Psychiatric Hospital Vrapce, Zagreb, Croatia; ^3^Faculty of Medicine, Josip Juraj Strossmayer University of Osijek, Osijek, Croatia; ^4^School of Medicine, University of Zagreb, Zagreb, Croatia; ^5^Department of Psychiatry, University Hospital Centre Zagreb, Zagreb, Croatia; ^6^Department of Psychopharmacology, Croatian Institute for Brain Research, School of Medicine, University of Zagreb, Zagreb, Croatia

**Keywords:** *BDNF*, Val66Met, C270T, BMI, plasma lipid levels, metabolic indices, PTSD

## Abstract

Posttraumatic stress disorder (PTSD) is a trauma and stressor related disorder that may develop after exposure to an event that involved the actual or possible threat of death, violence or serious injury. Its molecular underpinning is still not clear. Brain-derived neurotrophic factor (BDNF) modulates neuronal processes such as the response to stress, but also weight control, energy and glucose homeostasis. Plasma BDNF levels and a functional *BDNF* Val66Met (rs6265) polymorphism were reported to be associated with PTSD, as well as with increased body mass index (BMI) and dyslipidaemia in healthy subjects and patients with cardio-metabolic diseases, but these results are controversial. The other frequently studied *BDNF* polymorphism, C270T (rs56164415), has been associated with the development of different neuropsychiatric symptoms/disorders. As far as we are aware, there are no data on the association of *BDNF* Val66Met and C270T polymorphisms with metabolic indices in PTSD. Due to high rates of obesity and dyslipidaemia in PTSD, the aim of this study was to elucidate the association of *BDNF* Val66Met and C270T polymorphisms with BMI and lipid levels in veterans with PTSD. We hypothesized that *BDNF* variants contribute to susceptibility to metabolic disturbances in PTSD. The study included 333 Caucasian males with combat related PTSD, diagnosed according to DSM-5 criteria. Genotyping of the *BDNF* Val66Met and C270T polymorphisms was performed using the real-time PCR method. Results were analyzed using hierarchical multiple linear regression and the Mann–Whitney test, with *p*-value corrected to 0.005. The results showed that *BDNF* Val66Met and *BDNF* C270T polymorphisms were not significantly associated with BMI, total cholesterol, LDL-cholesterol, HDL-cholesterol or triglycerides. Although the *BDNF* C270T polymorphism was nominally associated only with HDL-cholesterol in veterans with PTSD, this significance disappeared after controlling for the effect of age. Namely, slightly higher plasma HDL values in T allele carriers, compared to CC homozygotes, were associated with differences in age. Our results, controlled for the critical covariates, revealed that *BDNF* Val66Met and C270T were not significantly associated with metabolic indices in veterans with PTSD and that these genetic variants do not contribute to susceptibility to metabolic disturbances in PTSD.

## Introduction

### Posttraumatic stress disorder

Posttraumatic stress disorder (PTSD) is a trauma- and stressor-related disorder (1) that develops in some, but not all individuals exposed to death, threatened death, actual or threatened serious injury, or actual or threatened sexual violence. Its molecular underpinning is still not clear. Various potential stressful events or traumas happen to people during their lifetime, but different populations vary in their exposure to traumatic events (2, 3). PTSD commonly co-occurs with different somatic disorders (4), such as cardiovascular, metabolic, dermatological, musculoskeletal, and pulmonary diseases (5).

### Metabolic disturbances in PTSD

Metabolic complications and components of the metabolic syndrome are associated with increased body mass index (BMI) values or weight gain, and with other clusters of physiological, biochemical, clinical, and metabolic factors that strongly increase the risk of type 2 diabetes and different cardiovascular diseases (6). These metabolic abnormalities include central obesity measured as waist circumference, dyslipidaemia (reduced HDL levels and increased triglyceride levels), high blood pressure, insulin resistance, as well as raised fasting plasma glucose. People with a sedentary life style, who do not exercise, consume a high fat diet and develop these metabolic abnormalities, have increased risk for cardiovascular and metabolic disorders (6).

Metabolic syndrome is frequent in PTSD (7–14). Metabolic complications include insulin resistance (15), higher mean triglycerides, higher blood pressure and fasting glucose levels, and lower HDL values in individuals with PTSD than in subjects from the general population (8). Young US veterans with PTSD had BMI values in the overweight range, higher than controls (15). Similarly, Croatian veterans with combat related PTSD had more frequently comorbid cardiometabolic disorders than control subjects (5), and this population had a high prevalence of obesity (16). Therefore, there is an urgent need for establishing the potential biomarkers of metabolic disturbances in PTSD (17).

### Brain-derived neurotrophic factor

The most abundant neurotrophin in the central nervous system is brain-derived neurotrophic factor (BDNF) and it modulates neuronal differentiation, synapse formation, survival, support and function of neurons, brain neurotransmission, proliferation, long-term potentiation, and synaptic growth in the central nervous system (18). Due to its localization and expression in the limbic system, brain regions that are involved in the regulation of fear and stress responses, and its modulatory role in dopaminergic, serotonergic and glutamatergic synthesis, metabolism, neuronal activity and release (18, 19), it is not surprising that BDNF is involved in the development of different neuropsychiatric disorders, including PTSD (19–21).

### *BDNF* genetic variants

In humans, BDNF is encoded by the *BDNF* gene which extends over 70 kb, located on chromosome 11, region p13–14 (22). There are hundreds of polymorphisms in the *BDNF* gene, however, the most frequently studied functional polymorphisms include the Val66Met single-nucleotide polymorphism (rs6265) in the coding exon (23), and the C270T single-nucleotide polymorphism (rs56164415), in the 5′-untranslated region (UTR) of the *BDNF* gene (24). The A (Met) allele, compared to the G (Val) allele, of the *BDNF* Val66Met is associated with disrupted cellular processing, trafficking and intracellular packaging of the pro-BDNF and reduced activity-dependent secretion of the mature BDNF (23, 25). The other polymorphism, *BDNF* C270T, in the *BDNF* 5'-non-coding region (26), may affect *BDNF* expression (27) and might lead to regionally specific quantitative BDNF disbalance in the brain (24). Consequently, both BDNF polymorphisms, *BDNF* Val66Met (19, 28) and *BDNF* C270T (24, 29) have been associated with different neuropsychiatric disorders.

### BDNF and PTSD

In patients with PTSD, inconsistent findings were reported regarding peripheral BDNF levels: blood levels were reported to be decreased (30), unchanged (31, 32), or increased (15, 33, 34). However, a meta-analysis did not confirm a significant association between BDNF levels and PTSD (35), which is in line with no significant relationship between cerebrospinal fluid BDNF levels and PTSD (36). There are conflicting findings on the relationship between *BDNF* genetic variants and PTSD (21, 37–40). The association between *BDNF* Val66Met and PTSD was confirmed in only one study (40), while other studies failed to detect a significant association (41–45). The association between PTSD and the other C270T polymorphism in the *BDNF* gene was investigated in two individual case-control studies, producing opposite results: positive (46) as well as no (45) association.

### BDNF and metabolic complications

The localization of BDNF in the hippocampus and the hypothalamus explains its moderating effects on energy metabolism, homeostasis, metabolic regulation (47, 48), weight control, fasting and feeding (49, 50). BDNF is located also in lungs, heart, spleen, gastrointestinal tract and liver, and it is involved in the development of cardiovascular and metabolic disorders, metabolic syndrome (51), and body weight gain (52, 53). In the blood, BDNF is stored mainly in platelets and released into plasma (54), but it is also synthesized and released from different cell types including cells from the cardiovascular system (55, 56), pancreatic beta cells, as well as cells from adipose tissue (57). BDNF exhibits an anorexigenic effect and suppresses food intake (48). A sedentary life style, overweight, obesity and lack of exercise are related to reduced BDNF signaling and increased activity of the sympathetic nervous system, decreased activity of the parasympathetic outflow, increased heart rate, blood pressure, elevated inflammation, and reduced gut motility (49).

There are conflicting data regarding an association of plasma or serum BDNF levels and metabolic indices. Reduced BDNF levels were detected in patients with type 2 diabetes (58), metabolic or coronary syndromes (57, 59, 60), non-obese and non-diabetic subjects with acute coronary syndrome (60), and in patients with angina pectoris (61). Increased BDNF levels were found in a population based study showing a positive association with increased risk for obesity, metabolic syndrome and coronary disease (51, 62). In contrast, in another study, subjects with or without metabolic syndrome had similar serum BDNF levels (63). A recent meta-analysis found lower BDNF levels associated with the presence of metabolic syndrome in healthy adult subjects (64). On the other hand, a longitudinal study including a large community-based cohort (65) detected higher serum BDNF levels significantly associated with lower risk of cardiovascular diseases and mortality, independent of markers of low-grade inflammation, BMI, physical activity, and depression.

*BDNF* gene variants were studied as risk factors for metabolic complications, such as BMI, dyslipidaemia, obesity, insulin resistance (66–68) and eating disorders (19). The A allele of the *BDNF* Val66Met polymorphism has been associated with higher BMI in adult women (69) and in young preschool children (70), but not in older healthy individuals (71). When these adult healthy groups were enlarged, a significant association was found between BMI categories and *BDNF* Val66Met (52, 72), since the A allele was more frequently found in the group with normal weight (52, 72–74). In conformation, obese subjects more frequently had the G allele of the *BDNF* Val66Met (73, 75). As far as we are aware, the reports on the association between the *BDNF* C270T polymorphism and BMI or plasma lipid levels in humans have not been studied.

In addition, to the best of our knowledge, there are no data on the association of *BDNF* Val66Met and *BDNF* C270T polymorphisms with metabolic indices in PTSD. Due to the high rates of both obesity and dyslipidaemia in PTSD, the aim of this study was to elucidate the association of *BDNF* Val66Met and *BDNF* C270T polymorphisms with BMI and lipid levels in veterans with PTSD. We hypothesized that *BDNF* variants might contribute to susceptibility to metabolic disturbances in PTSD and that these *BDNF* metabolic-risk variants might be more frequently present in patients with PTSD in comparison to healthy subjects or other diagnostic categories.

## Methods

### Participants

The study included 333 male veterans with combat related PTSD, with median Clinician Administered PTSD Scale (CAPS) scores of 86 (range 68–102). They were all unrelated Caucasian subjects of Croatian origin. The diagnosis of current and chronic PTSD was done using SCID based on DSM-5 criteria (1). Participants were sampled consecutively in the University Psychiatric Hospital Vrapce, Zagreb, from September 2015 to June 2017. They were exposed to similar potentially traumatic events during the Homeland war in Croatia. Inclusion criteria were in- and out-patients aged 38–77 years. Exclusion criteria were: drug abuse, alcohol dependence or pathophysiological changes in the liver, such as fibrosis, sclerosis, cirrhosis and malignant liver disease [alcoholic liver cirrhosis (K70.3), alcoholic liver fibrosis and sclerosis (K70.2) and hepatocellular carcinoma (C22.0), according to ICD-10], schizophrenia, bipolar disorder, adult ADHD, Alzheimer's disease (according to DSM-5 criteria), current or recent (previous 3 months) use of lipid-lowering agents, antihypertensive and antidiabetic medication. The study was approved by the Ethics Committee of the University Psychiatric Hospital Vrapce, Zagreb, Croatia, and was carried out in accordance with the Helsinki declaration (1975), as revised in 1983. All patients have signed informed consent prior to study procedures.

### Anthropological measures

Height of subjects wearing no shoes was measured with a meter to the nearest 0.5 cm; whereas body weight of subjects was measured with a digital scale to the nearest 0.1 kg. BMI was calculated as ratio of weight (kg) over height (m^2^).

### Measurements of the metabolic indices

Blood samples were collected between 7:30 a.m. and 8:00 a.m. after overnight fasting. Total cholesterol (normal values < 5 mmol/l) was determined with cholesterol oxidase-phenol aminophenazone method and the absorbance read on a Siemens Dimension Xpand analyser. Triglycerides (normal values < 1.7 mmol/l), LDL (normal values < 3 mmol/l), and HDL (normal values >1.2 mmol/L) were determined using the enzymatic-colorimetric assay and were analyzed with Siemens Dimension Xpand analyser.

### Genotyping

Genomic DNA was isolated from peripheral blood using a salting out method (76). *BDNF* Val66Met (rs6265) and C270T (rs56164415) were determined with TaqMan® Genotyping Assays (Applied Biosystems, Foster City, CA, USA) following the manufacturer's protocol on an Applied Biosystems® 7300 Real-Time PCR System apparatus. The 10 μL reaction volume contained around 20 ng of DNA. Assay IDs were C_11592758_10 for rs6265 and C_89097201_10 for rs56164415. Around 10% of randomly selected samples were genotyped again as a quality control for genotyping assays.

Minor allele frequency (MAF) for *BDNF* Val66Met in our sample was 19% (A allele), which is in accordance with the MAF of 20% (A allele) in the European population (77). In our European sample, MAF for *BDNF* C270T was 17% (T allele), while 1000 Genomes reports much smaller frequency of T allele (6%) in this population.

Since there were only 6 AA genotype carriers (of the *BDNF* Val66Met) and 3 TT genotype carriers (of the *BDNF* C270T) in the whole sample, we assessed only the dominant model for the *BDNF* Val66Met: A carriers (AA + AG) vs. GG homozygous genotype, and the dominant model for the *BDNF* C270T: T carriers (TT + TC) vs. CC homozygous genotype (78, 79).

### Statistical analysis

All data regarding lipid levels, BMI and age failed to reach normal distribution (Kolmogorov-Smirnov test). The data were expressed as median, 25th (Q1) and 75th (Q3) percentile after excluding outliers. Outliers were determined as values that lie below Q1–1.5 interquartile range (IQR) or above Q3+1.5 IQR. Hierarchical multiple linear regression was used to determine possible effect of age, smoking, BMI, *BDNF* Val66Met, and *BDNF* C270T on each metabolic parameter after excluding outliers. The data were evaluated with the non-parametric Mann-Whitney test for each metabolic parameter using Sigma Stat 3.5 (Jandell Scientific Corp. San Raphael, California, USA). For determination of the linkage disequilibrium (LD) between *BDNF* Val66Met and C270T loci, we used Haploview software v. 4.2 (80). Loci are considered to be in high LD if the D' coefficient is >0.80 and logarithm of odds (LOD) ≥ 2 (80, 81). In our study these two loci were not in high LD since the D′ coefficient was < 0.80 (D′ = 0.36; LOD = 0.32). Therefore, we did not perform haplotype analysis. Due to multiple (*N* = 10) comparisons (testing the association of 2 single nucleotide polymorphisms (SNPs) with 5 metabolic indices = 10), a Bonferroni correction was performed and *p*-value was set to *p* = 0.005. Before the study G^*^Power 3 Software (82) was used to determine the required sample size and actual statistical power. For hierarchical multiple linear regression, with *p* = 0.005; medium effect size = 0.25; and power (1 – β) = 0.800; number of predictors = 5; the required sample size was 142. For the Mann–Whitney test, with *p* = 0.005; medium effect size = 0.15; and power (1 – β) = 0.800; the required sample size was 217. Since the study included 333 participants in the beginning, and 294–311 participants after the removal of outliers, it had adequate sample size and statistical power to detect significant differences among the groups.

## Results

### Clinical data

Demographic and clinical data, including age, levels of total, HDL and LDL cholesterol, triglyceride levels, and BMI, of veterans with PTSD are presented in Table 1. Initially, the study enrolled 333 subjects. However, after removal of the outliers for each metabolic parameter (values that were lower than Q1–1.5 IQR or higher Q3+1.5 IQR in our sample), there were *N* = 294 participants for HDL (removed HDL values ≤ 0.5 and HDL ≥ 2.0), *N* = 316 for BMI (removed BMI values ≤ 19); *N* = 315 for cholesterol (removed cholesterol values ≥8.4), *N* = 311 for LDL (removed LDL values ≥5.7 and LDL ≤ 0.2) and *N* = 303 for triglycerides (removed triglyceride values ≥3.8), respectively.

**Table 1:** Demographic and clinical data of veterans with PTSD.

	**N**	**Median (25th; 75th)**	**Min–Max**
Age (years)	333	56 (51; 63)	38–77
TC (mmol/L)	315	5.20 (4.50; 6.00)	2.90–8.10
HDL (mmol/L)	294	1.20 (1.10; 1.40)	0.80–1.80
LDL (mmol/L)	311	2.80 (2.40; 3.60)	0.90–5.60
TG (mmol/L)	303	1.60 (1.30; 2.20)	0.60–3.70
BMI (kg/m^2^)	316	28.03 (25.85; 30.45)	20.32–35.92

*Results are presented as median and 25th (Q1) and 75th (Q3) percentiles. N is number of subjects after removing outliers. PTSD, posttraumatic stress disorder; TC, total cholesterol; HDL, high density lipoprotein cholesterol; LDL, low density lipoprotein cholesterol; TG, triglycerides; BMI, body mass index*.

### The effect of age, smoking, BMI, and *BDNF* polymorphisms on metabolic parameters

Hierarchical multiple linear regression was performed in order to assess the influence of various independent variables, such as age, smoking, BMI, and *BDNF* Val66Met and *BDNF* C270T on different metabolic parameters (dependent variables) (Table 2). It was designed in the following way: for all metabolic indices, except BMI, the first step included age, BMI, and smoking status as dependent variables, and the second step *BDNF* Val66Met genotype (dominant model) and *BDNF* C2790T genotype (dominant model). For BMI, age, and smoking were entered as dependent variables in the first step, while both genotypes were added in the second one. Hierarchical multiple regression revealed age as the only significant (*p* < 0.001) variable influencing HDL and triglycerides, while other variables were not significantly associated with metabolic indices due to Bonferroni corrected significance (*p* = 0.005).

**Table 2:** Hierarchical multiple linear regression showing variables influencing different metabolic parameters.

		**TC**	**HDL**	**LDL**	**TG**	**BMI**
Step 1	Model summary	adj*R*^2^ = −0.006; Δ*R*^2^ = 0.005; *F* = 0.449; *p* = 0.718	adj*R*^2^ = 0.065; Δ*R*^2^ = 0.076; *F* = 7.355; *p* < 0.001;	adj*R*^2^ = −0.004; Δ*R*^2^ = 0.006; *F* = 0.621; *p* = 0.602;	adj*R*^2^ = 0.042; Δ*R*^2^ = 0.052; *F* = 5.057; *p* = 0.002;	adj*R*^2^ = −0.003; Δ*R*^2^ = 0.004; *F* = 0.577; *p* = 0.652;
	Age	β = −0.051; *p* = 0.387	β = −0.246; *p* < 0.001	β = 0.039; *p* = 0.508	β = 3.406; *p* = 0.001	β = 0.015; *p* = 0.804
	BMI	β = 0.034; *p* = 0.564	β = 0.129; *p* = 0.029	β = −0.070; *p* = 0.237	β = −0.012; *p* = 0.835	–
	Smoking	β = 0.032; *p* = 0.583	β = 0.008; *p* = 0.896	β = −0.015; *p* = 0.798	β = 0.116; *p* = 0.050	β = −0.061; *p* = 0.299
Step 2	Model summary	adj*R*^2^ = −0.006; Δ*R*^2^ = 0.006; *F* = 0.637; *p* = 0.672;	adj*R*^2^ = 0.065; Δ*R*^2^ = 0.007; *F* = 4.820; *p* < 0.001;	adj*R*^2^ = −0.008; Δ*R*^2^ = 0.003; *F* = 0.537; *p* = 0.746;	adj*R*^2^ = 0.040; Δ*R*^2^ = 0.005; *F* = 3.337; *p* = 0.006;	adj*R*^2^ = −0.010; Δ*R*^2^ = 0.000; *F* = 0.300; *p* = 0.878;
	Age	β = −0.023; *p* = 0.714	β = −0.216; *p* = 0.001	β = 0.037; *p* = 0.552	β = 2.922; *p* = 0.004	β = 0.014; *p* = 0.820
	BMI	β = 0.033; *p* = 0.573	β = 0.128; *p* = 0.030	β = −0.070; *p* = 0.209	β = −0.012; *p* = 0.842	–
	Smoking	b = 0.035; *p* = 0.557	β = 0.009; *p* = 0.877	β = −0.015; *p* = 0.806	β = 0.113; *p* = 0.055	β = −0.061; *p* = 0.302
	BDNF Val66Met	β = −0.020; *p* = 0.735	β = 0.007; *p* = 0.908	β = −0.050; *p* = 0.398	β = 0.062; *p* = 0.298	β = −0.013; *p* = 0.828
	BDNF C270T	β = −0.084; *p* = 0.180	β = −0.088; *p* = 0.162	β = 0.016; *p* = 0.803	β = 0.048; *p* = 0.434	b = −0.004; *p* = 0.953

*A carriers (AA + AG) combined genotypes of the BDNF Val66Met; T carriers (TT + TC) combined genotypes of the BDNF C270T; BDNF, brain derived neurotrophic factor; BMI, body mass index; TC, total cholesterol; HDL: high density lipoprotein cholesterol; LDL, low density lipoprotein cholesterol; TG, triglycerides*.

### *BDNF* Val66Met and *BDNF* C270T polymorphisms and metabolic indices

Table 3 shows that metabolic parameters did not differ significantly between individuals with PTSD subdivided into A carriers (AA+AG) vs. homozygous GG genotype carriers of *BDNF* Val66Met, as well as between T carriers (TT+CT) vs. CC genotype carriers of the *BDNF* C270T. Namely, no significant differences between A carriers and GG homozygotes of the *BDNF* Val66Met polymorphism, were detected in the BMI values (*p* = 0.979), plasma concentrations of total (*p* = 0.933), HDL (*p* = 0.829), and LDL (p = 0.146) cholesterol, as well as triglycerides (*p* = 0.409). In the case of the *BDNF* C270T polymorphism, similar results were observed. The Mann Whitney test revealed that BMI (*p* = 0.822), plasma concentrations of total (*p* = 0.738) and LDL (*p* = 0.290) cholesterol, as well as triglyceride (*p* = 0.092) levels were similar in T carriers and CC homozygotes. However, HDL levels differed nominally (*p* = 0.006) between T carriers and CC homozygotes of the *BDNF* C270T polymorphism. Since this association was not confirmed using hierarchical multiple linear regression, the observed difference in HDL levels was presumably associated with the age differences between these groups.

**Table 3:** The metabolic parameters of veterans with PTSD subdivided according to the *BDNF* Val66Met and *BDNF* C270T genetic variants.

	**SNP**	***BDNF*** **Val66Met**		***BDNF*** **C270T**	
	**Genotype**	**A carriers**	**GG carriers**	**T carriers**	**CC carriers**
BMI (kg/m^2^)	Median	27.75	27.91	27.77	28.06
	Percentile 25th; 75th	26.26; 30.11	25.69; 30.47	25.86; 30.47	26.03; 30.45
	Min; Max	20.32; 35.92	20.76; 35.71	20.76; 33.91	21.26; 35.92
	Statistics	*U* = 10581.00; *p* = 0.979		*U* = 10743.50; *p* = 0.822	
TC (mmol/L)	Median	5.20	5.20	5.20	4.90
	Percentile 5th; 75th	4.50; 5.70	4.50; 6.10	4.50; 6.10	4.50; 6.00
	Min; Max	2.90; 8.10	2.90; 7.90	3.00; 8.10	2.90; 7.90
	Statistics	*U* = 10485.00; *p* = 0.933		*U* = 10555.00; *p* = 0.738	
HDL (mmol/L)	Median	1.20	1.20	1.20	1.20
	Percentile 25th; 75th	1.10; 1.40	1.10; 1.40	1.10; 1.50	1.10; 1.30
	Min; Max	0.80; 1.80	0.80; 1.80	0.80; 1.80	0.80; 1.80
	Statistics	*U* = 9065.50; *p* = 0.829		*U* = 7561.50; *p* = 0.006	
LDL (mmol/L)	Median	3.00	2.80	2.80	2.90
	Percentile 25th; 75th	2.40; 3.60	2.30; 3.50	2.20; 3.60	2.40; 3.60
	Min; Max	0.90; 5.40	0.90; 5.60	0.90; 5.60	0.90; 5.40
	Statistics	*U* = 9157.00; *p* = 0.146		*U* = 9658.00; *p* = 0.290	
TG (mmol/L)	Median	1.60	1.60	1.55	1.60
	Percentile 25th; 75th	1.30; 2.10	1.30; 2.20	1.20; 2.10	1.30; 2.30
	Min; Max	0.60; 3.70	0.60; 3.70	0.60; 3.70	0.60; 3.70
	Statistics	*U* = 9165.50; *p* = 0.409		*U* = 8637.00; *p* = 0.092	

*Results are presented as median and 25th (Q1) and 75th (Q3) percentiles after excluding outliers. A carriers (AA + AG) of the BDNF Val66Met; GG (GG homozygous genotype) carriers of the BDNF Val66Met; T carriers (TT + TC) of the BDNF C270T; CC carriers (CC homozygous genotype) of the BDNF C270T; BDNF: brain derived neurotrophic factor; BMI, Body mass index; HDL, high density lipoprotein cholesterol; LDL, low density lipoprotein cholesterol; PTSD, posttraumatic stress disorder; TC, total cholesterol; TG, triglycerides*.

Therefore, as age was a significant (*p* < 0.001) variable that affects HDL and triglycerides in the regression model, and HDL levels were nominally (*p* = 0.006) different between T carriers and CC homozygotes, to further evaluate this age-related, but also *BDNF* C270T -related influence, patients were additionally subdivided into 3 age groups. Since the age range of the patients was 38–77, we subdivided participants into approximately 13-year age groups, i.e., into individuals between the ages of 38 and 51 (*N* = 76 for HDL, and *N* = 74 for triglycerides after removing outliers), ages 52–65 (*N* = 162 for HDL, *N* = 171 for triglycerides), and ages 66–77 (*N* = 56 for HDL and *N* = 58 for triglycerides). The Mann-Whitney test demonstrated no significant (*p* > 0.005) differences between *BDNF* Val66Met A vs. GG genotype carriers, and between *BDNF* C270T T vs. CC genotype carriers in HDL and triglyceride levels (Table 4) in each age group. These results revealed that these two polymorphisms were not significantly associated with HDL and triglyceride levels in any of the age groups. Nevertheless, median HDL values (Table 4) showed a decline (as evidenced by the multiple linear regression analysis: β = −0.243) with increased age in both T and CC genotype carriers.

**Table 4:** The HDL and TG values in different age groups of veterans with PTSD subdivided according to the *BDNF* Val66Met and *BDNF* C270T genetic variants.

	**Age group**	**38–51 years**		**52–65 years**		**66–77 years**	
***BDNF* Val66Met**	**Genotype**	**A carriers (*N* = 32)**	**GG carriers (*N* = 44)**	**A carriers (*N* = 56)**	**GG carriers (*N* = 106)**	**A carriers (*N* = 15)**	**GG carriers (*N* = 41)**
HDL (mmol/L)	Median	1.30	1.30	1.20	1.20	1.20	1.10
	Percentile 25^th^; 75^th^	1.10; 1.50	1.10; 1.50	1.00; 1.30	1.10; 1.30	1.10; 1.20	1.00; 1.20
	Min; Max	0.80; 1.80	0.80; 1.80	0.80; 1.80	0.80; 1.80	0.90; 1.40	0.80; 1.80
	Statistics	***U*** = 690.00; ***p*** = 0.754		***U*** = 2740.50; ***p*** = 0.660		***U*** = 161.50; ***p*** = 0.149	
***BDNF* Val66Met**	**Genotype**	**A carriers (*N* = 32)**	**GG carriers (*N* = 42)**	**A carriers (*N* = 58)**	**GG carriers (*N* = 113)**	**A carriers (*N* = 15)**	**GG carriers (*N* = 43)**
TG (mmol/L)	Median	1.50	1.60	1.60	1.60	1.50	2.20
	Percentile 25th; 75th	1.30; 1.80	1.20; 1.80	1.30; 2.20	1.20; 2.20	0.90; 2.20	1.50; 3.20
	Min; Max	0.60; 2.70	0.60; 3.70	0.60; 3.70	0.60; 3.70	0.70; 3.70	0.70; 3.70
	Statistics	***U*** = 639.50; ***p*** = 0.721		***U*** = 3135.50; ***p*** = 0.790		***U*** = 139.00; ***p*** = 0.042	
***BDNF* C270T**	**Genotype**	**T carriers (*N* = 38)**	**CC carriers (*N* = 38)**	**T carriers (*N* = 52)**	**CC carriers (*N* = 110)**	**T carriers (*N* = 4)**	**CC carriers (*N* = 52)**
HDL (mmol/L)	Median	1.30	1.30	1.20	1.20	1.10	1.10
	Percentile 25th; 75th	1.10; 1.50	1.10; 1.50	1.10; 1.50	1.10; 1.20	1.00; 1.45	1.00; 1.20
	Min; Max	0.80; 1.80	0.80; 1.80	0.80; 1.80	0.80; 1.80	0.90; 1.80	0.80; 1.80
	Statistics	***U*** = 723.00; ***p*** = 0.992		***U*** = 2333.00; ***p*** = 0.054		***U*** = 106.00; ***p*** = 0.963	
***BDNF* C270T**	**Genotype**	**T carriers (*N* = 34)**	**CC carriers (*N* = 40)**	**T carriers (*N* = 56)**	**CC carriers (*N* = 115)**	**T carriers (*N* = 4)**	**CC carriers (*N* = 54)**
TG (mmol/L)	Median	1.55	1.50	1.55	1.60	1.55	2.20
	Percentile 25th; 75th	1.30; 1.80	1.20; 1.80	1.20; 2.00	1.30; 2.30	1.35; 1.90	1.50; 2.70
	Min; Max	0.80; 3.70	0.60; 3.40	0.60; 3.60	0.60; 3.70	1.20; 2.20	0.70; 3.70
	Statistics	***U*** = 632.00; ***p*** = 0.463		***U*** = 2945.50; ***p*** = 0.365		***U*** = 66.50; ***p*** = 0.212	

*Results are presented as median and 25th (Q1) and 75th (Q3) percentiles after excluding outliers. A carriers (AA + AG) of the BDNF Val66Met (rs6265); GG (GG homozygous genotype) carriers of the BDNF Val66Met; T carriers (TT + TC) of the BDNF C270T; CC carriers (CC homozygous genotype) of the BDNF C270T; BDNF, brain derived neurotrophic factor; HDL, high density lipoprotein cholesterol; PTSD, posttraumatic stress disorder; TG, triglycerides*.

## Discussion

The results of this study revealed that in a homogeneous sample of middle-aged Caucasian (Croatian origin) veterans with combat related PTSD: (1) *BDNF* Val66Met and *BDNF* C270T polymorphisms were not significantly associated with BMI or plasma lipid levels; (2) the presence of one or two T alleles of *BDNF* C270T polymorphism was related to slightly (nominally) higher HDL cholesterol values; but this association disappeared after controlling for the influence of age.

In the present study, veterans had chronic and current PTSD, and moderate PTSD symptoms, as revealed by their mean CAPS scores of 86 (range 68–102) (83). Their lipid levels were either slightly higher (total cholesterol and triglycerides) or within the normal laboratory range (i.e., for HDL and LDL cholesterol levels). Data from the literature regarding lipid levels in PTSD are inconsistent (84). Similar HDL and LDL cholesterol levels were found in different groups of Caucasian veterans with PTSD of the same origin (85), or in Japanese civilians with PTSD, who were victims of sarin poisoning (86). Further, HDL levels did not differ between civilian participants with and without PTSD (87, 88). Similarly, PTSD was associated with unchanged LDL cholesterol levels (89, 90). Other studies found dyslipidaemia, i.e., increased cholesterol, triglyceride, and LDL cholesterol levels in PTSD (88, 91–93). In contrast to our data, some studies have detected lower HDL cholesterol levels in PTSD (89, 90, 94), or similar triglyceride levels in large groups of community-living adults with or without PTSD (87). Further, cholesterol, LDL cholesterol and triglyceride levels did not differ between participants with PTSD compared to non-PTSD individuals (94). Dyslipidaemia and increased BMI are risk factors for various cardiovascular diseases and myocardial infarction in all subjects (6), especially individuals with PTSD (4, 8, 10, 13). Therefore, careful monitoring of these patients is warranted to prevent development of cardiometabolic disorders. The current investigation included patients with PTSD without cardiovascular diseases and the exclusion criteria included taking cardiovascular drugs or statins, but their mean BMI values of almost 28 suggest that the majority of participants were overweight. This is in line with data obtained with the different groups of Croatian veterans with PTSD (16) but also with data from a Croatian population sample (16), and with findings from young military US veterans with PTSD, who were all overweight (15). All these findings suggest that overweight, obesity and dyslipidaemia are becoming health risks in PTSD, but also global health problems in the general population. Various risk factors such as different diagnoses and sizes of the groups, civilian or military individuals, diet, food, sedentary life style and physical activity, age, exercise, alcohol dependence, and smoking might affect BMI and lipid levels. As this study did not intend to compare BMI and lipid levels between individuals with PTSD and control subjects, we may conclude that our results on the lipid levels and BMI are in line with most of the cited data.

There was one study showing positive association (40) and others reporting no associations (41–45) between *BDNF* Val66Met and PTSD. In our previous study that included different groups of war veterans (42), the frequency of the *BDNF* Val66Met genotypes did not differ between veterans with or without PTSD. Therefore, the present study with other groups of veterans with PTSD did not evaluate this possible association and did not include control subjects. The explanation for these inconsistent findings might be sought in the frequency of trauma exposure, traumatic load, early traumatic experience and stressful life events, age, and other study conditions among the participants (21).

We did not confirm our hypothesis that *BDNF* variants contributed to susceptibility to metabolic disturbances in PTSD. In our study *BDNF* Val66Met polymorphism was not significantly associated with metabolic indices in Caucasian veterans with PTSD. Namely, BMI values, as well as plasma total cholesterol, triglycerides, HDL and LDL cholesterol levels did not differ significantly in veterans with PTSD subdivided into carriers of the A allele (AA+AG) or the GG homozygous genotype of *BDNF* Val66Met polymorphism. As there are no such data in the literature, we might presume that this study is the first to show no association of *BDNF* Val66Met variants with BMI in combat veterans with PTSD. In line with our data, BMI was not significantly different in A carriers compared to GG genotype carriers of the *BDNF* Val66Met in elderly healthy Chinese subjects (95), or in elderly healthy individuals who were followed longitudinally (71). However, there are conflicting results in the literature regarding this polymorphism and BMI in control subjects. Opposing data were reported showing that the A allele was associated with higher BMI in healthy adult subjects (96), adult women (69), and in young preschool children (70). On the other hand, there are also data showing that A carriers were more frequently found in the group with lower BMI in a large Korean epidemiological cohort (74), in a large cohort of male Boston Puerto Rican subjects (67), adult healthy Caucasian women (73, 75), healthy control male and female subjects from the same population of Caucasian subjects of Croatian origin (52, 72), or in children and adolescents (97). A recent meta-analysis confirmed only one *BDNF* SNP, rs925946, significantly related to obesity and BMI in large groups of healthy subjects (98). Discrepancies in the results could be explained by the different diagnostic groups (PTSD vs. controls), ethnic background (19, 99, 100), age, gender, living environment, diet, lifestyle, and smoking (95). Since recently a strong LD between *BDNF* rs6265 and rs10501087 was reported, suggesting that these two SNPs are dependent genetic markers associated with BMI (98), in future studies this *BDNF* SNP, rs10501087, should be also evaluated.

Our study did not detect any significant association between *BDNF* Val66Met and lipid levels in PTSD. There are no findings in the literature regarding the association between *BDNF* Val66Met and lipid levels in PTSD. However, in volunteers without kidney and cardiovascular diseases, no association was found between *BDNF* Val66Met polymorphism and serum TG and HDL cholesterol levels (63). This finding agrees with the results from our study, since our veterans with PTSD, who did not have any cardiovascular or metabolic diseases, had similar plasma lipid levels when subdivided into carriers of different *BDNF* Val66Met variants. In children and adolescents from the general population, the *BDNF* Val66Met polymorphism was not associated with any of the lipid indices (97). In contrast to our study and cited (63, 97) findings, the presence of one or two A alleles of the *BDNF* Val66Met was significantly associated with lower HDL cholesterol levels and higher risk for obesity in old and very old Chinese healthy subjects (95). In that particular study subjects carrying the A allele had elevated triglyceride levels, but reduced HDL cholesterol levels compared to the GG genotype carriers (95). The differences between studies are in the diagnosis (PTSD vs. normal controls) and in the age of included subjects (i.e., Peng's study included three groups: age ≥90; age 60–77; and age 60–75, while our veterans were on average 56 years old). To control for the effect of age, in our study participants with PTSD were subdivided into different age groups (age range 38–51, 52–65, and 66–77 years), and their plasma HDL and triglyceride levels (that were significantly affected by age in the hierarchical regression analysis) did not differ between *BDNF* Val66Met A vs. GG genotype carriers and between *BDNF* C270T T vs. CC genotype carriers. In addition, the observed differences between studies might be due to ethnicity (Chinese vs. Caucasian subjects), since there were significant ethnic related differences in the frequency of the *BDNF* Val66Met genotypes, especially the frequency of the AA genotype, between Asian and Caucasian subjects (100).

Unlike *BDNF* Val66Met, the *BDNF* C270T polymorphism has only recently gained attention in studies of neuropsychiatric disorders (101). The T allele of the *BDNF* C270T polymorphism was reported to be a risk factor for PTSD (46), schizophrenia (29), bulimia nervosa (102), and amyotrophic lateral sclerosis (24), but not for Alzheimer's disease (78). The T allele prevalence in our study was 17% and it was the same as the T allele frequency in a Chinese sample with PTSD (46). This frequency differs significantly from the T allele frequency in our healthy Caucasian subjects (6%, unpublished data) and in the general East Asian population [6%, (77)] or in other diagnostic groups. Namely, in Caucasian patients with schizophrenia this frequency was around 8% (29), while in Caucasian patients with bulimia nervosa and control subjects it was around 5% (102). Higher prevalence of the T allele in our Caucasian subjects and in Chinese subjects with PTSD (46) compared to control subjects (102) and patients with schizophrenia (29) or bulimia (102), might be an outcome of different clinical diagnoses (PTSD vs. schizophrenia vs. bulimia nervosa/controls). This is also supported by the fact that no obvious ethnic differences in *BDNF* C270T genotype frequency were found in a recent meta-analysis (78) and reported by The 1000 Genomes Project Consortium (77), with the exception of South Asian populations (India, Bangladesh, Pakistan) where the T allele is present in 29% of population.

The *BDNF* C270T polymorphism was related to PTSD in one study, since the T allele frequency was significantly higher in a group with sporadic PTSD compared to a control group (46). Another study did not find a significant association, but this trial comprised only 96 subjects with PTSD (45). Since in our study we have not compared the frequency of the *BDNF* C270T genotypes in PTSD vs. controls, we cannot confirm or reject this finding.

This investigation has not found a significant association between *BDNF* C270T and BMI, total cholesterol, LDL cholesterol and triglyceride levels in veterans with PTSD. A slight association between *BDNF* C270T and HDL cholesterol in veterans with PTSD was detected, as carriers of the T alleles had nominally higher HDL cholesterol levels than CC carriers. This significance did not survive a Bonferroni correction, and was not confirmed by hierarchical multiple regression and further testing. Only HDL and triglyceride levels were affected by age. Other metabolic indices were not associated with age, smoking, *BDNF* Val66Met or *BDNF* C270T polymorphisms. To further evaluate these findings, HDL and triglyceride levels were evaluated in participants subdivided into 13-year age groups, i.e., into individuals in the age range from 38 to 51, from 52 to 65, and from 66 to 77 years, although we are aware of the limited power due to the stratification of data into smaller groups. HDL and triglyceride levels did not differ between *BDNF* Val66Met A allele carriers vs. GG genotype carriers, and between *BDNF* C270T T allele carriers vs. CC genotype carriers. As both *BDNF* C270T T allele carriers and CC genotype carriers showed reduced median HDL values in older age, these results confirmed that HDL was affected by age and not by the presence of the T allele.

There are no other data on the association of this *BDNF* C270T polymorphism and HDL cholesterol in PTSD, and there are no data showing either a positive association or no association between *BDNF* C270T polymorphism and BMI, total cholesterol, LDL cholesterol and triglyceride levels in PTSD. Although this was the first study to evaluate a link between BDNF C270T and metabolic indices in PTSD, no associations were found between either of the variants and these metabolic indices. One study reported that *BDNF* C270T polymorphism was not associated with BMI in patients with anorexia nervosa and bulimia nervosa (103), while another found a significant association between the T allele of the *BDNF* C270T polymorphism and lower BMI in bulimia nervosa (102). The discrepancies between these and our study might be explained by the differences in diagnoses (PTSD vs. eating disorders).

Our results did not confirm our hypothesis that *BDNF* Val66Met and C270T variants contribute to susceptibility to metabolic disturbances in PTSD. Lower HDL cholesterol values were detected in older individuals with PTSD, but were not associated with any of the *BDNF* Val66Met and C270T genotypes. Routine monitoring of individuals with PTSD, in terms of HDL cholesterol levels, might prevent possible cardiovascular events including myocardial infarction, that are frequent in PTSD (5, 17, 84).

Some limitations need to be considered in interpreting these results: the study included only Caucasian PTSD veterans, and all participants were males. Therefore, we could not compare our data with those in control subjects, and we could not evaluate possible ethnic and gender related differences. In this study we included only the two SNPs (*BDNF* Val66Met and C270T polymorphisms), so additional *BDNF* gene polymorphisms should be included in future studies, such as those evaluated in healthy control subjects: *BDNF* rs12291063 polymorphism associated with BMI and obesity (104), *BDNF* rs10767664 polymorphism associated with BMI, weight, fat mass, waist circumference, LDL cholesterol and total cholesterol (105), or *BDNF* rs925946 polymorphism associated with BMI (98), or GWAS data. Data on physical activity and eating habits were not collected. A significant limitation of this study is a lack of replication sample.

Advantages of the present study are the use of a fairly large group of veterans with PTSD, sampled from the same center, and in the fact that diagnosis and screening for PTSD was done by psychiatrists using SCID and CAPS. We also took into account alcohol dependence and use of statins and cardiovascular drugs, we removed outliers, carefully monitored the confounders by a hierarchical multiple linear regression, used a homogeneous group of Caucasians of Croatian origin with similar combat trauma exposure and ensured that the study had the required sample size and statistical power.

## Conclusion

This is a first report showing that variants of the *BDNF* Val66Met and *BDNF* C270T polymorphisms were not associated with BMI or plasma lipid levels in veterans with PTSD. Our findings suggest that *BDNF* Val66Met and C270T variants do not contribute to susceptibility to metabolic disturbances in PTSD. Other metabolic indices and other *BDNF* and other gene variants should be evaluated as metabolic risk factors in PTSD in further larger multi-ethnic studies, including females, larger sample-sizes, with well-matched controls.

## Author contributions

NP developed the original idea. LT, MK, MNP, DSS, and GNE managed the experimental work, collected blood samples, isolated DNA and did the BDNF rs6265 and rs56164415 genotyping, and processed data for analysis. LT performed the statistical analysis. SU, OK, and ZKP explained the research goals and described protocol in details to the patients; explained the inclusion/exclusion criteria, insured participant adherence for the participation in the study, motivated, selected, diagnosed, evaluated and sampled patients with PTSD. NP did the data analysis and interpretation. NP and MS wrote the first and the final draft of the article. DSS wrote part of the article and revised the article. MNP and DSS did the proof reading of the manuscript. All authors contributed to the final version of the manuscript. All authors have revised the article and approved the final article.

### Conflict of interest statement

The authors declare that the research was conducted in the absence of any commercial or financial relationships that could be construed as a potential conflict of interest.
